# Difficulty in Assessing Quality of Life Outcomes in a Fluctuating Disease: A Hypothesis Based on Gastroparesis

**DOI:** 10.1155/2009/396190

**Published:** 2009-10-15

**Authors:** Vic Velanovich

**Affiliations:** Division of General Surgery, K-8, Henry Ford Hospital, 2799 West Grand Boulevard, Detroit, MI 48202, USA

## Abstract

An underlying assumption of quality of life outcome research is that after some intervention a “steady-state” of quality of life is reached which can be identified as an endpoint, and, hence, the “outcome.” However, in some disease processes, no so such steady-state is reached. The hypothesis presented is that a disease process with a waxing and waning course will make it difficult to determine a quality of life endpoint. After clinical observation, a pilot study of patients with either diabetic or idiopathic gastroparesis with gastric neurostimulation their records were reviewed to identify the number of clinic visits, the number of clinic visits in which the patients were asymptomatic, much improved, improved, no change, worse, or much worse. These changes were defined as “transitions.” A “transition ratio” was calculated by dividing the number of transitions by the number of clinic visits. Preliminary results showed that of 32 patients, the median number of clinic encounters was 8 (1–35), and the median number of transitions 4 (0–22). The average transition ration was 0.56 ± 0.31. In the case of gastroparesis, over half of all clinical encounters were associated with a transition. The implication of the hypothesis and preliminary finding suggests a difficulty to identify when the symptomatic endpoint was reached. Other methods to assess the effects of treatment in such a disease process are required to fully understand the effects of treatment on quality of life.

## 1. Introduction

**Figure 1:**
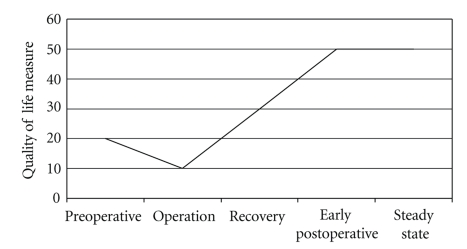
Hypothetical quality of life measurement after an operation for a disease. After initial fall in quality of life related to the operation, as the patient recovers, quality of life will improve to a level better than the patient's preoperative status.

The purpose of quality of life research is to explore the effects of disease and its treatment on a variety of patient-centered experiences. This is done using a range of methods, primarily with some type of quality of life instruments. In clinical medicine, one of the most active areas of research is related to the quality of life effects of a specific treatment. In this setting, the usual study design is to have a group of patients with the disease of interest complete one or more instruments prior to the intervention, then the patients receive the intervention (with or without a comparison group not receiving the intervention), then at some arbitrary time, the instrument is readministered [1]. An underlying assumption, albeit frequently unstated, is that at the point of post treatment reassessment, the patient has reached the maximal benefit (or detriment) of the treatment. This can be thought of as a “steady-state” (Figure 1). Theoretically, this steady-state is the quality of life outcome of the intervention.

## 2. An Hypothesis

In practice, however, identifying this steady-state may be problematic. In most quality of life studies, quality of life is assessed at some arbitrary temporal endpoint, say, 3 months, 6 months, 1 year, and so forth, The assumption made by these researchers is that whatever effect the intervention has made on quality of life would have been reached by that time. For many diseases and intervention, this may be true. However, it is also clear that not all disease processes fit this model. That is to say, these diseases have a natural waxing and waning symptomatic and quality of life natural history. Examples of such diseases are inflammatory bowel disease and rheumatoid arthritis. Therefore, at the point the instrument is administered (a “good day” or a “bad day”), will affect the quality of life data obtained. The hypothesis presented is that in such disease processes which have a natural history of a waxing and waning symptomatic course, such an arbitrary endpoint may be misleading as to the overall effect of the treatment on the disease. An example of one such disease, gastroparesis [2], and how its natural symptomatic volatility affects the assessment of the gastric neurostimulation is presented as a pilot study.

## 3. Evaluation of the Hypothesis

This study was approved by the institutional review board of the Health Ford Health System. The Enterra device (Medtronic, Inc., Minneapolis, Minn, USA) provides electrical stimulation to the stomach with the purpose of decreasing the symptoms of gastroparesis. It is approved for use by the United States Food and Drug Administration under their Humanitarian Device Exemption for patients with diabetic and idiopathic gastroparesis. Patients were followed up every two to four weeks until maximal symptomatic improvement was reached, then they were to complete quality of life instruments. 

A retrospective review of all clinical encounters of post-treatment patients treated for diabetic or idiopathic gastroparesis was done. The medical record was reviewed for age, gender, length of followup, and number of post-treatment clinical encounters. A clinical encounter included all outpatient visits specifically related to gastroparesis or management of the neurostimulator, or an inpatient hospitalization at which time a consultation was requested for gastroparesis or management of the neurostimulator. All patients were followed prospectively and during each clinical encounter, patients were asked as to their symptoms of gastroparesis. Patients rated their symptoms as much worse, worse, no change, improved, much improved, or asymptomatic. Any change in symptoms, whether improved or worsened, was defined as a “transition.” The concept here is that when a steady-state is reached, there will be few, if any, transitions. A “transition ratio” was calculated as transition ratio = number of clinical encounters with a transition/total number of clinical encounters. Both the concepts of transitions and transition ratio are novel to the thinking of the natural history of gastroparesis.

In order to have a better understanding of symptomatic variability over time, plots of the change of symptom severity for individual patients were graphed. Arbitrarily, if a patient reported symptom improvement, then his/hers symptom level rose by 1; if much improved, then by 2; if worsened, it decreased by 1; much worse, by 2. If a patient reported that he was asymptomatic, then a score of 10 was given. If the patient reported no change in symptoms, then the score was kept at the same level as his/her previous visit.

## 4. Empirical Data

A number 32 patients were included in the study: 23 were female; the mean age of all patients was 42 ± 14 years. The median followup was 8 months (range: 1–37 months). The median number of clinical encounters was 8 (range: 1–35). The median number of clinical transitions was 4 (range: 0–22). The mean transition ratio was 0.56 ± 0.31.

**Figure 2:**
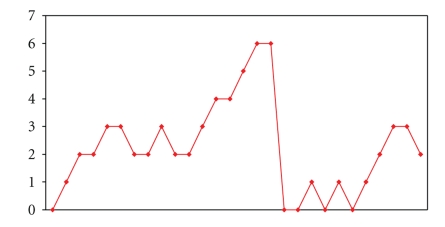
A gastroparesis patient with a great deal of symptomatic variability from clinical encounter to clinical encounter.

**Figure 3:**
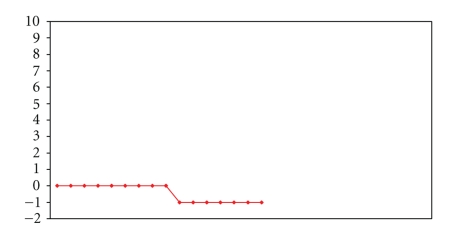
A patient whose symptoms did not change until clinical encounter number 8, then worsened, with no change after that.

Figures 2 and 3 present individual patients variation in symptom severity from clinical visit to clinic visit.

## 5. Implications of the Hypothesis and Discussion

The implications of this hypothesis and preliminary findings is that choosing an arbitrary temporal endpoint in diseases with variability in symptoms and quality of life may over or under estimate the overall effect of treatment. The importance of symptomatic variability lies in determining when the “outcome” of the treatment is reached. If an arbitrary temporal endpoint is used, say 1 month, 1 year, and so forth, then whether the patient is having a “good day” or a “bad day” will affect how the patient answers the items of whatever instrument is used. Therefore, the outcome measured at this point may not be fully reflective of the patient experience.

A counter argument is that if the treatment is truly effective in improving symptoms and quality of life in aggregate, the summary statistics (mean, median, etc.) should be better than control subjects' or the patients' baseline summary statistics. It is interesting, though, that in a cross-over trial of gastric neurostimulation, patients more frequently preferred the device to be on (i.e., providing electrical stimulation) than off, even though the symptom severity score used showed no statistically significant difference [3]. Although one of the potential explanations for this finding is that the instrument used was not sensitive enough to detect change in the symptoms important to the patient, the other explanation is that the variability in symptoms was so great that the standard error of the mean was to wide to be of statistical significance. Yet, the patients themselves, over the time period that the device was on, felt that there was overall improvement despite the symptomatic variability. Therefore, summary statistics in disease processes with “natural” variation in symptom severity and quality of life may show, in statistical terms, a type II error—that is to say, not accepting that a true difference exist when in fact one does [4]. One can easily see this is the case in gastroparesis with in about one-half of clinical encounters patients reported either improvement or worsening of their symptoms from the previous encounter. Even, this statistic does not accurately reflect individual patient symptomatic variability, as evidence by plotting the change in symptoms from one clinical encounter to the next for individual patients (Figures 2 and 3).

Several approaches have been developed to address longitudinal quality of life data [5]. Examples of these include analyzing the area of curve, graphical presentations, tabular presentations, or statistical modeling techniques [5]. Although it is not the purpose of this study to assess the advantages or disadvantages of each technique, all do require repeated, multiple administrations of whatever instrument is used. Obviously, as the number of administrations is increased, the chance of encountering missing data is also increased.

In summary, this hypothesis and preliminary results demonstrate that patients suffering from idiopathic or diabetic gastroparesis have temporally related variability in their symptoms. Therefore, the choice of when a symptom severity or quality of life instrument is administer may affect that data that is obtained depending on whether it was a good day or a bad day for the patient. Such data may not be truly reflective of the patient experience and may lead to affect statistical analysis. New approaches need to be developed for such diseases.
